# Supplementary material for: Diagnosis, clinical characteristics, and outcomes of COVID-19 patients from a large healthcare system in northern New Jersey
Source: Sci Rep. 2021 Feb 23;11:4389. doi: 10.1038/s41598-021-83959-7 (PMC7902820; doi:10.1038/s41598-021-83959-7)
Supplement: Supplementary file 1 — Supplementary Information. [file 41598_2021_83959_MOESM1_ESM.pdf]

Diagnosis, Clinical Characteristics, and Outcomes of COVID-19 Patients from A Large Healthcare System in Northern New Jersey

Yanan Zhao[1,2#], Marcus H. Cunningham[1], Jose R. Mediavilla[1], Steven Park[1], Sean Fitzgerald[1], Hee Sang Ahn[3], Xiangyang Li[3], Caixin Zhan[3], Tao Hong[3,4,5], Gary Munk[2,5,7], Kar Fai Chow[3,6], David S. Perlin[1#]

[1]Center for Discovery and Innovation, Hackensack Meridian Health, Nutley, NJ, USA

[2]Department of Medical Sciences, Hackensack Meridian School of Medicine, Nutley, NJ, USA

[3]Molecular Diagnostic Laboratory, Department of Pathology, Hackensack University Medical Center, Hackensack, NJ, USA

[4]Microbiology Laboratory, Department of Pathology, Hackensack University Medical Center, Hackensack, NJ, USA

[5]Department of Pathology, Hackensack Meridian School of Medicine, Nutley, NJ, USA

[6]Core Laboratory, Department of Pathology, Hackensack University Medical Center, Hackensack, NJ, USA

[7]Clinical Virology Laboratory, Department of Pathology, Hackensack University medical Center, Hackensack, NJ, USA

# Address correspondence to Yanan Zhao, Ph.D. yanan.zhao@hmh-cdi.org, phone: 201-880-3503 or David S. Perlin, Ph.D. david.perlin@hmh-cdi.org, phone: 201-880-3500

SUPPLEMENTARY MATERIAL

CDI Enhanced COVID-19 Test

The CDI virus test is a hybrid diagnostic panel, by cherry-picking the best components from the WHO-adopted test [1] and the CDC test (https://www.fda.gov/media/134922/download). As a result, the CDI test consists of E assay from the WHO test, N2 and RP assay from the CDC test. E assay is conserved for all SARS-like coronaviruses, while N2 assay is specific for SARS-CoV-2. The primers and probes for the CDI diagnostic panel are listed on **Table S1**.

Table S1. Summary of CDI Enhanced COVID-19 Test

Assay	Detection purpose	Primer/Probe	Sequences (5'-3')
E	conserved detection of SARS-like coronaviruses	E-F	ACAGGTACGTTAATAGTTAATAGCGT
		E-R	ATATTGCAGCAGTACGCACACA
		E-P	FAM-ACACTAGCCATCCTTACTGCGCTTCG-BHQ1
N2	specific detection of SARS-CoV-2	2019-nCoV_N2-F	TTACAAACATTGGCCGCAAA
		2019-nCoV_N2-R	GCGCGACATTCCGAAGAA
		2019-nCoV_N2-P	FAM-ACAATTTGCCCCCAGCGCTTCAG-BHQ1
RP	internal control, detection of human Rnase-P gene	RP-F	AGATTTGGACCTGCGAGCG
		RP-R	GAGCGGCTGTCTCCACAAGT
		RP-P	FAM-TTCTGACCTGAAGGCTCTGCGCG-BHQ1

Real-time RT-PCR

The One Step PrimeScript™ RT-PCR Kit (Perfect Real Time) (Takara) or the SensiFAST™ Probe No-ROX One-Step Kit (Bioline) was used for RT-PCR master mix preparation, depending on the availability of the reagent. Assay setup incorporated published primer/probe concentrations from the original testing panel with the manufacturer's recommendation for the remainder components. Specifically, if TaKara kit was used, a total volume of 20 µl reaction contained 10 µl of one step RT-PCR Buffer III, 0.4 µl of PrimeScript RT enzyme Mix II, 0.4 µl of TaKaRa Ex Taq HS (5U/µl), 1.2 µl of primer/probe premix for CDC test (or 2.4 µl of primer/probe premix for WHO test), and 5 µl of RNA/TNA template. A universal thermal profile was used for all assays as 42°C for 5 min for reverse transcription, followed by 95°C for 10 sec then 45 cycles of 95°C for 5 sec and 58°C for 20 sec. If the Bioline kit was the option, assays were still set up in a total volume of 20 µl reaction comprising 10 µl of 2x SensiFAST Probe No-ROX One-Step Mix, 0.2 µl of reverse transcriptase, 0.4 µl of RiboSafe RNase Inhibitor, 1.2 µl of primer/probe

premix for CDC test (or 2.4 µl of primer/probe premix for WHO test), and 5 µl of RNA/TNA template. Thermal profile was changed to 45°C for 10 min for reverse transcription, followed by 95°C for 2 min then 45 cycles of 95°C for 5 sec and 58°C for 20 sec. All real-time testing was performed using the Bio Molecular System mic qPCR cycler, and micPCR software v2.8.0 was used for data analysis. All FDA required positive and negative controls were included in parallel for all assays in every single run.

Reference

1 Corman, V. M. *et al.* Detection of 2019 novel coronavirus (2019-nCoV) by real-time RT-PCR. *Euro surveillance : bulletin Europeen sur les maladies transmissibles = European communicable disease bulletin* **25**, doi:10.2807/1560-7917.Es.2020.25.3.2000045 (2020).
